# Congenital bilateral folds in Descemet’s membrane with high astigmatism

**DOI:** 10.3205/oc000134

**Published:** 2020-02-27

**Authors:** Steffi Vande Walle, Cathérine Cassiman

**Affiliations:** 1 Department of Ophthalmology, University Hospitals of Leuven, Leuven, Belgium

**Keywords:** folds in Descemet’s membrane, astigmatism, prolonged labor

## Abstract

We present the case of a boy with congenital bilateral folds in Descemet’s membrane, causing high astigmatism and myopia. There are multiple causes of folds and tears in Descemet’s membrane. In our case, the most likely origin is the mother’s prolonged labor, although a severe car accident of the mother at the gestational age of 27 weeks as the cause of these folds cannot be entirely excluded.

## Introduction

Congenital bilateral folds in Descemet’s membrane can have multiple causes, ranging from congenital glaucoma over corneal dystrophy to trauma. Early diagnosis and treatment is important to prevent severe amblyopia due to pronounced astigmatism.

## Case description

A boy aged 1 year and 7 months was seen at our clinic after referral for refractive errors with Plusoptix. Retinoscopy under cyclopegia revealed: –14.5 diopters with an oblique astigmatism of –2 diopters on the right eye and –9.50 diopters with an oblique astigmatism of –4 diopters on the left eye. Fundoscopy and intra-ocular pressure were normal. Glasses were prescribed. An A-scan showed symmetric axial lengths of the left and right eye. B-scan echography showed a thicker lens on the right eye, which could explain the anisomyopia. A few months later, glasses were adapted based on new values on retinoscopy: right eye –10.5(–8x180°), left eye –3(–6x140°). Due to high anisomyopia, patching of the left eye was initiated daily for 2 hours.

At the age of 2 years and 2 months, glasses were adapted to –7.00 (–9.00x40°) for the right eye and –5.00 (–7.00x140°) for the left eye. Patching was continued.

Due to the unexplained high refractive error and the difficult clinical examination of this child, a detailed examination under general anesthesia with a keratometry, automatic refractor and pachymetry, detailed examination of the anterior segment and dilated fundoscopy were performed. Results are shown in Table 1 (Tab. 1). Upon retro-illumination, bilateral parallel oblique folds in Descemet’s membrane were found. Gonioscopy was not performed.

The general history of this boy reports a serious car accident of the mother during pregnancy at the gestational age of 27 weeks. All follow-up controls afterwards were reassuring. The patient was born on the postmenstrual age of 35 weeks and 3 days. Labor was induced because of preterm premature rupture of membranes (PPROM). An urgent caesarean section was performed because of fetal distress. There were no other neonatal problems. The patient was diagnosed with platelet dysfunction at the age of 1 year on the occasion of recurrent hematomas caused by minor trauma. An extended screening for underlying metabolic disease could not reveal any abnormalities. There were no other health problems. Familial history for any eye disorders was negative.

## Discussion

The main cause of tears or folds in Descemet’s membrane is congenital glaucoma. Congenital glaucoma can cause breaks in Descemet’s membrane due to raised intraocular pressure with stretching of the cornea. The breaks typically have a horizontal orientation and are called Haab’s striae. Children with congenital glaucoma present with epiphora, photophobia and blepharospasm. Most cases are bilateral. Clinical examination reveals high intra-ocular pressure, dysgenesis of the trabeculum, corneal haze and raised optic disc cupping which may regress once intraocular pressure has normalized. There are some cases described in the literature where congenital glaucoma has arrested spontaneously [1], [2], [3]. Our patient did not show any signs of congenital glaucoma. His striae were oblique, while in congenital glaucoma they are typically horizontal. Corneal diameters and intra-ocular pressure were within normal limits (Table 1 (Tab. 1)).

Posterior polymorphous corneal dystrophy (PPCD) is an autosomal dominant inherited corneal dystrophy. Clinical examination shows isolated vesicles or broad horizontal bands with scalloped edges at the posterior surface of the cornea. Most patients are asymptomatic, and the condition is discovered by chance. Our patient had high myopic astigmatism along the axis of the striae, which did not fit the fact that most patients with PPCD are asymptomatic. The condition is inherited in an autosomal dominant pattern, while our patient’s parents did not show any corneal abnormalities. PPCD bands typically have a horizontal orientation.

Bhagat et al. [4] described a case of breaks in Descemet’s membrane due to non-accidental injury. Our patient had a history of multiple hematomas. Because of suspicion of battered child, he was allocated to a hospital by court for further examination. Blood samples revealed a platelet dysfunction as the cause of recurrent hematomas and the case was closed. We do not suspect the corneal folds being caused by non-accidental trauma.

At the gestational age of 27 weeks, the mother had been involved in a serious car accident. She had worn a seat belt at the crash, but we consider it highly unlikely that this has caused the bilateral Descemet folds, since the amniotic fluid would have cushioned the foetus.

Folds in Descemet’s membrane are a well-described entity following a complicated forceps-assisted delivery. The first report dates of 1895 [5]. They are unilateral, parallel with a vertical or oblique orientation due to horizontal compression of the eye between the orbit and the blades of the forceps. The folds are typically left-sided due to the position of the foetus at birth with left occiput first. Prolonged labor can also induce these folds. In our patient’s case, labor had been induced because of PPROM, but the delivery had not progressed. Due to foetal distress, a caesarean section had eventually been performed.

Intra-operative optical coherence tomography (OCT) of the cornea could have been of benefit to evaluate the corneal folds in this patient, since there is insufficient cooperation to perform this examination in the consultation setting. This tool was not available in our center at the time of presentation of the patient. OCT could show us the exact extent of the folds and their effect on the entire structure of the cornea.

## Conclusion

Prolonged labor seems to have been the most likely cause of these bilateral oblique folds in Descemet’s membrane. However, pressure of the seat belt during the car crash cannot be entirely excluded.

## Notes

### Competing interests

The authors declare that they have no competing interests.

### Informed consent

The parents of the patient gave consent to publish photographs of his ocular lesions and to publish this case report.

## Figures and Tables

**Table 1:**
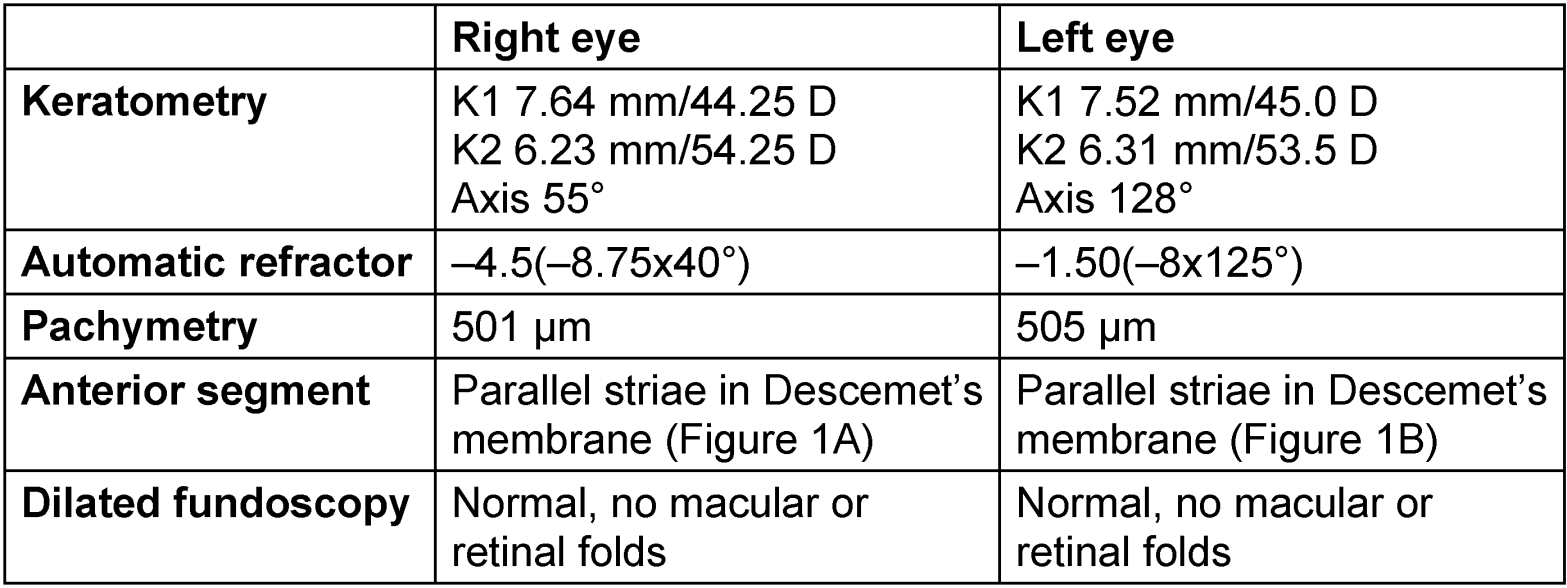
Results of the examination under general anesthesia
